# Applying inter-rater reliability to improve consistency in classifying PhD career outcomes

**DOI:** 10.12688/f1000research.21046.2

**Published:** 2020-04-28

**Authors:** C. Abigail Stayart, Patrick D. Brandt, Abigail M. Brown, Tamara Dahl, Rebekah L. Layton, Kimberly A. Petrie, Emma N. Flores-Kim, Christopher G. Peña, Cynthia N. Fuhrmann, Gabriela C. Monsalve

**Affiliations:** 1 University of Chicago, Chicago, IL, USA; 2 University of North Carolina at Chapel Hill, Chapel Hill, NC, USA; 3 Vanderbilt University, Nashville, TN, USA; 4 Emory University and the Georgia Institute of Technology, Atlanta, GA, USA; 5 UCI School of Biological Sciences, Irvine, CA, USA; 6 University of Massachusetts Medical School, Worcester, MA, USA; 7 University of California, San Francisco, San Francisco, CA, USA

**Keywords:** workforce development, higher education, career outcomes, STEM education, career taxonomy

## Abstract

**Background:** There has been a groundswell of national support for transparent tracking and dissemination of PhD career outcomes. In 2017, individuals from multiple institutions and professional organizations met to create the Unified Career Outcomes Taxonomy (UCOT 2017), a three-tiered taxonomy to help institutions uniformly classify career outcomes of PhD graduates. Early adopters of UCOT 2017, noted ambiguity in some categories of the career taxonomy, raising questions about its consistent application within and across institutions.

**Methods:** To test and evaluate the consistency of UCOT 2017, we calculated inter-rater reliability across two rounds of iterative refinement of the career taxonomy, classifying over 800 PhD alumni records via nine coders.

**Results:** We identified areas of discordance in the taxonomy, and progressively refined UCOT 2017 and an accompanying Guidance Document to improve inter-rater reliability across all three tiers of the career taxonomy. However, differing interpretations of the classifications, especially for faculty classifications in the third tier, resulted in continued discordance among the coders. We addressed this discordance with clarifying language in the Guidance Document, and proposed the addition of a flag system for identification of the title, rank, and prefix of faculty members. This labeling system provides the additional benefit of highlighting the granularity and the intersectionality of faculty job functions, while maintaining the ability to sort by - and report data on - faculty and postdoctoral trainee roles, as is required by some national and federal reporting guidelines. We provide specific crosswalk guidance for how a user may choose to incorporate our suggestions while maintaining the ability to report in accordance with UCOT 2017.

**Conclusions:** Our findings underscore the importance of detailed guidance documents, coder training, and periodic collaborative review of career outcomes taxonomies as PhD careers evolve in the global workforce. Implications for coder-training and use of novice coders are also discussed.

## Introduction

Recently, national conversation has highlighted the need for greater institutional transparency in reporting career outcomes of graduate and postdoctoral alumni. In addition to fulfilling institutional reporting requirements, public sharing of outcomes data provides information to prospective and current trainees about the broad career paths that leverage their PhD training. Outcomes data also informs the evolution of graduate and postdoctoral education practices and policies. In response to the current lack of career outcome visibility, organizations and funding agencies have made concerted efforts to encourage and support institutional commitment to public sharing of career outcomes data. Many groups have called for institutional transparency in career outcomes for PhD-trained scientists, including, but not limited to: the National Institutes of Health (NIH) ^1^, the Future of Bioscience Graduate and Postdoctoral Training (FOBGAPT) conferences I & II ^2, 3^, Rescuing Biomedical Research (RBR) ^4, 5^, Future of Research (FoR) ^6^, the UW-Madison Workshop ^7^, the National Institute of Health Broadening Experiences in Scientific Training (BEST) Consortium ^8, 9^, the Coalition for Next Generation Life Science (NGLS Coalition) ^10^, Council of Graduate Schools (CGS) ^11^, the Association of American Universities (AAU) ^12^, the Association of American Medical Colleges (AAMC) ^13^, and the National Academy of Sciences ^14, 15^. Many institutions are now publicly sharing their alumni career outcomes data on websites and in publications ^8, 16– 19^. However, a factor that has impacted the sharing of outcomes data has been a confusion over which career outcomes taxonomy is being displayed in which context. The absence of a unified language for reporting the career outcomes of graduate and postdoctoral alumni perpetuates an environment in which universities, policy makers, and prospective trainees are unable to compare career outcomes on a local, regional, or national level.

In Spring 2017, 14 schools within the BEST Consortium formed a working group to design a taxonomy of career descriptions that could be used across institutions to consistently describe the career outcomes of PhD and postdoctoral alumni trained in the biological and biomedical sciences. The working group used the Science Careers myIDP ^20^ career categories as its starting point for the taxonomy. This work was subsequently incorporated into a collaborative effort led by RBR, which convened national stakeholders, including experts in graduate training and career development from AAMC, the NIH, the AAU, and academic institutions both internal and external to the BEST Consortium. The resultant three-tiered Unified Career Outcomes Taxonomy (UCOT 2017) was developed with the aim of creating a publicly available, standardized, and valid measure that was vetted by experts at the national level. It was first released online by the BEST Consortium ^21^, and reports of the meeting, including additional recommendations for data collection, were reported by RBR, AAMC, and BEST ^5, 13, 22, 23^. The process of developing UCOT 2017 and its associated recommendations was an example of cross-organizational communication, collaboration, and compromise. Several institutions, including those of the Coalition for Next Generation Life Science, have moved forward in adopting UCOT 2017 as a first step toward tracking and comparing career outcomes ^24^.

The UCOT 2017 has three tiers of classification: Workforce Sector, Career Type, and Job Function. While the categories in each tier were deemed sufficiently broad to describe the primary career trajectories of PhDs in the biological sciences, the breadth of those definitions permitted significant room for discordant interpretation. Based on our early attempts to utilize UCOT 2017, we were concerned that the breadth of the definitions could obscure the nuances of the complex career taxonomy and result in unreliable and inconsistent application by individuals *(i.e., coders)* across different institutions responsible for coding the outcomes data. We were particularly concerned about coding categories in the Job Function tier which provides for a granular refinement of the specific skill sets and/or credentials required for employment within that function. For example, a Program Director title at a University could have very different job responsibilities compared to a person of the same title in a life science consulting firm. Without a reliable, reproducible, and robust schema for the classification of career outcomes, cross-site comparisons would have limited validity and the value of national reporting would be diluted ^25^. We believed it to be crucial to test the reliability of UCOT 2017 and to uncover any inconsistencies in the application of the career taxonomy by different coders. In this study, we identified taxonomic categories that resulted in low concordance ( *i.e., low inter-rater reliability*) among coders and iteratively modified the career taxonomy until all three tiers met or exceeded reliability standards.

## Methods

Institutional Review Board (IRB) approvals for this project were obtained from each institution that provided alumni records to the study ( *Emory University: IRB# H13506; Vanderbilt University School of Medicine: IRB# 180315; University of North Carolina, Chapel Hill: IRB# 14-0544*). Institutional consent processes for data collection and consent were reviewed separately as participating institutions of the NIH BEST consortium (variants included an electronic information sheet, inclusion of a standardized NIH BEST consortium-wide informed consent paragraph, and approval as exempt or non-human subjects research designations with no consent required). In all cases, IRB approvals included the stipulation to remove any identifiable information from participant data. In accordance with this requirement, only de-identified data are reported and all subject identification numbers have been reassigned to protect participant identities.

### Development of the guidance document for UCOT 2017

Four coders from the BEST Consortium ( *PDB, TD, AMB, CAS*) conducted a preliminary application of UCOT 2017 to code 2,587 graduate student alumni records from across their respective institutions ( *data not reported here due to IRB data-sharing limitations*). This preliminary application of UCOT 2017 revealed several areas of confusion. Therefore, prior to beginning Experiment 1, this group agreed upon initial clarifications to UCOT 2017, therein generating “UCOT experimental version 1” (UCOT Exp1). Two of the group members ( *TD and CAS*) created a Draft Guidance Document to specifically address confusing categories that were discovered during preliminary use of UCOT 2017 and were anticipated to result in discordant interpretation during Experiment 1. The Draft Guidance Document included some elaboration on how to classify faculty, entrepreneurs, and postdocs and other types of training positions.

**Figure 1.**
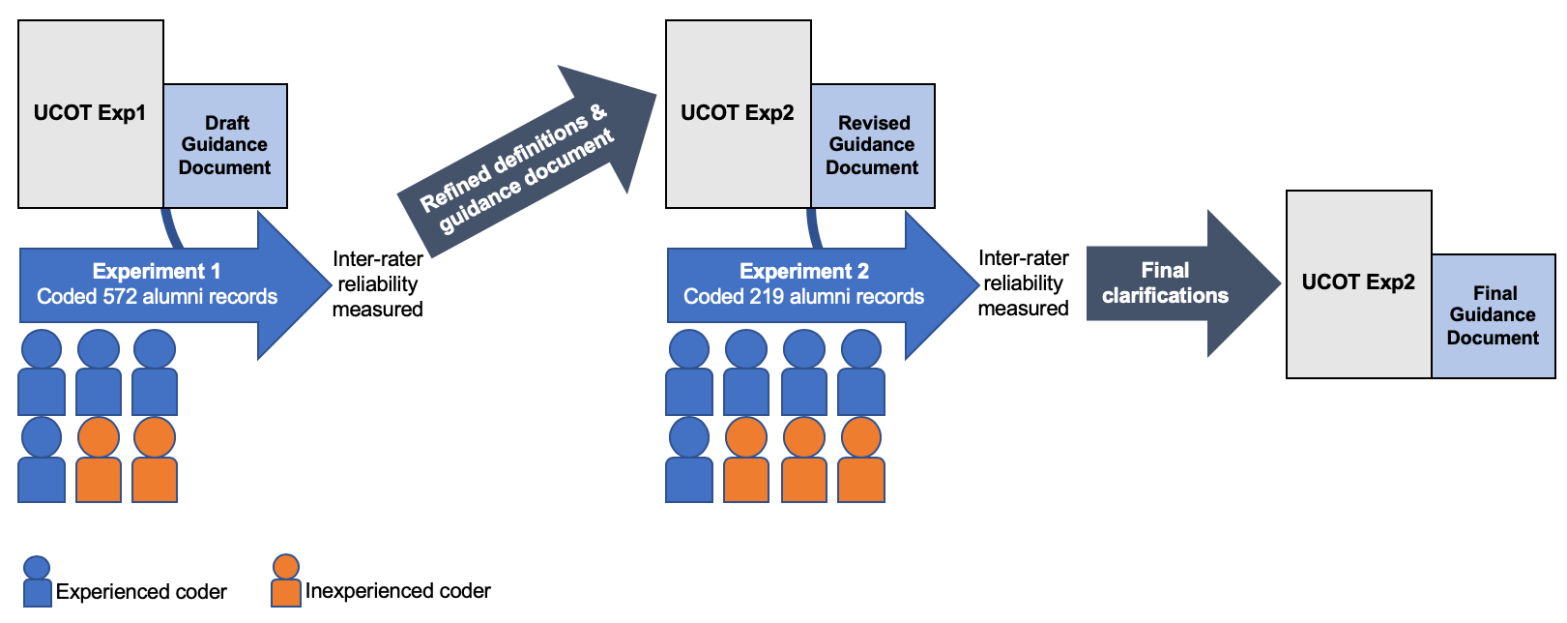
Experimental design to assess inter-rater reliability of the Unified Career Outcomes Taxonomy 2017. In Experiment 1, six coders comprising four experienced coders *(blue)* and two inexperienced coders *(orange)* classified 572 PhD alumni records from three different institutions using UCOT Exp1 and the Draft Guidance Document. After measurements of inter-rater reliability were determined, the working group convened to refine the career taxonomy definitions and guidance materials, generating experimental version 2 (UCOT Exp2) and the Revised Guidance Document. The reliability of UCOT Exp2 and the Revised Guidance Document were then assessed using a new data set of 219 PhD alumni records. In Experiment 2, coding was performed by five experienced coders ( *blue*) and three new inexperienced coders ( *orange*). After classification of the records, inter-rater reliability was measured. The group convened again to review the results and introduce further clarifications to the Revised Guidance Document, therein creating the Final Guidance Document.

***Experimental overview.*** Figure 1 provides an overview of our experimental design. Using sets of alumni records from three universities, our overall experiment was composed of two stages: a first round of coding using a modified version of the 2017 Unified Career Outcomes Taxonomy (UCOT Exp1), and a second round of coding (with some returning coders and some new coders) using UCOT Exp2. UCOT Exp2 is a modified version of UCOT Exp1 that was refined based on inter-rater reliability analyses and coder feedback following the first round. After each round, inter-rater reliability was calculated for each tier of the career taxonomy and taxonomic categories were identified that caused particular discordance across coders.

### Coders

The team involved in Experiment 1 consisted of individuals who had previous experience with the career taxonomy, including four individuals who had participated in the original BEST working group and were responsible for generating UCOT Exp1 (two were also present at the national meeting associated with developing UCOT 2017). The Experiment 1 team also included two inexperienced coders who were new to both the career taxonomy and the experiment. The team of coders involved in Experiment 2 included five experienced coders who were involved in Experiment 1 and three inexperienced coders who had no previous involvement in the project. Beyond the coders from Exp 1 who took on the task, additional coders were recruited from the NIH BEST Consortium Program Directors/Staff during NIH BEST meetings, consortium calls, and conversations. In total, nine coders participated in one or both stages of this study. At the time of this study, all nine were university administrators in professional roles focused on career development for PhD scientists, with experience ranging from six months to fifteen years in the field. Eight of the nine coders had doctoral degrees in the biological or social sciences; the team consisted of seven women and two men.

### Selection of alumni records for experimental analysis

The pool of alumni records from which the experimental data sets were selected included the 2,587 graduate student alumni previously coded by Vanderbilt University, Emory University, and UNC Chapel Hill. This sample of records represented all alumni data available since the inception of each respective institutional umbrella program (e.g., Vanderbilt’s Interdisciplinary Graduate Program in Biological and Biomedical Sciences, Emory’s Graduate Division of Biological and Biomedical Sciences, and UNC’s Biological and Biomedical Sciences), excluding records that could not be verified or located. The sample was intended to establish a combined pool large enough to provide representative distribution across job categories for a robust dataset to code. Each record was composed of a unique record number, current job title, current employer, LinkedIn profile or other job-related URL, and graduation date. Postdoctoral alumni were not included in the data set due to inconsistent collection of career outcomes for this population across institutions.

**Figure 2.**
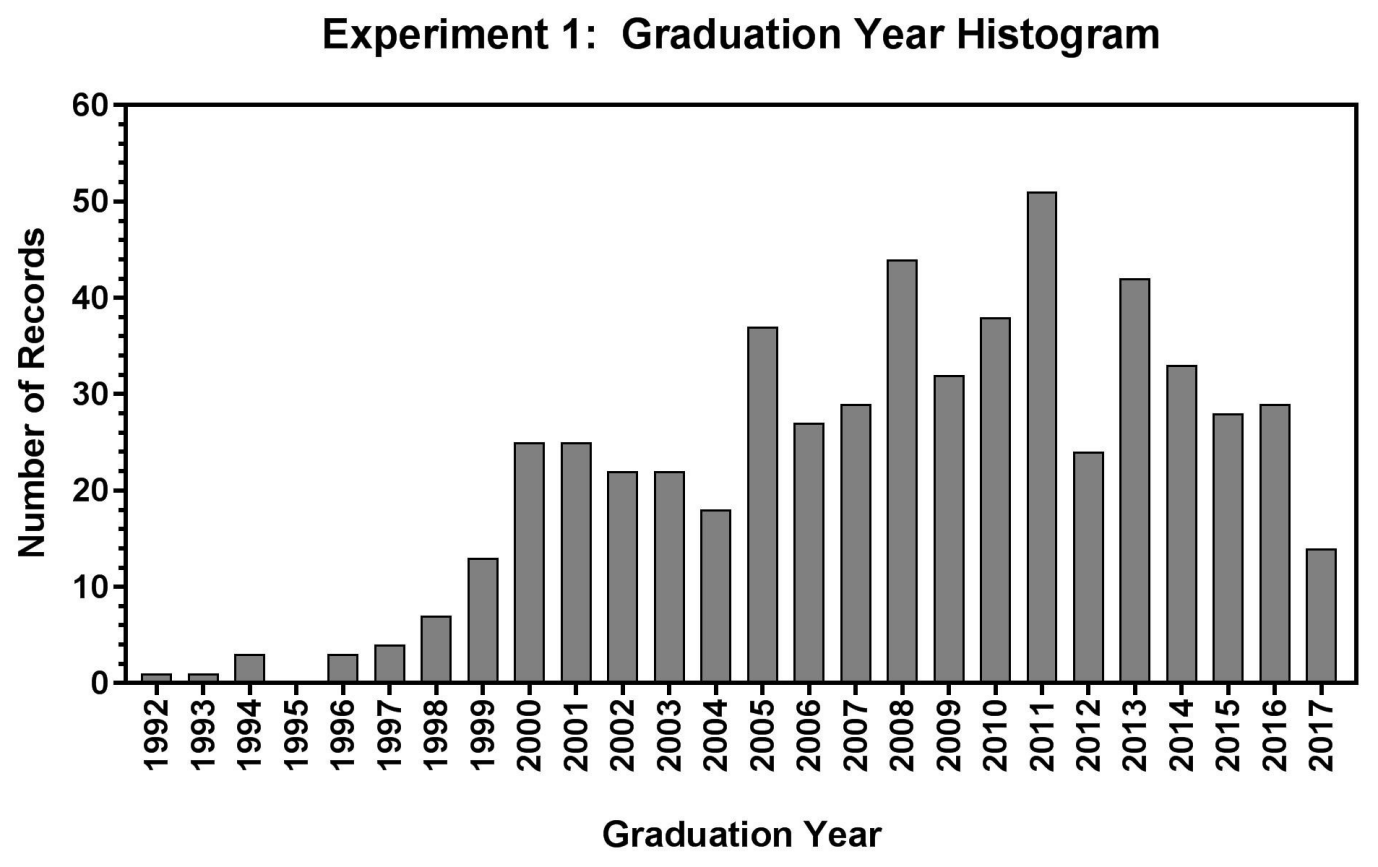
Histogram of graduation years for Experiment 1 records. Distribution of graduation years for all records coded in Experiment 1 using UCOT Exp1.

The data set coded in Experiment 1 contained 572 alumni records, including 185 records from UNC Chapel Hill, 192 from Vanderbilt, and 195 from Emory. Women comprised 47% of the dataset, men comprised 28%, and 25% of the alumni records were of unknown gender. Figure 2 shows the distribution of alumni records according to year of graduation. For a detailed description of how alumni records were selected for inclusion in Experiment 1, please refer to *Extended data:* S4.

**Figure 3.**
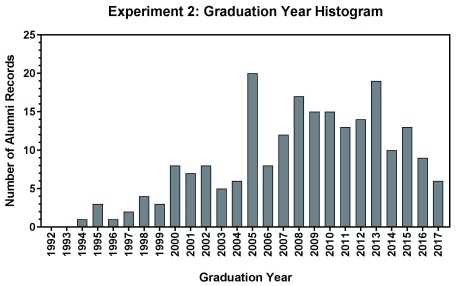
Histogram of graduation years for Experiment 2 records. Distribution of graduation years for all records coded in Experiment 2 using UCOT Exp2.

The data set coded in Experiment 2 contained 219 alumni records. A minimum representative sample across each Job Function category was used to select the number of records; where possible, three records per job function were chosen from each institution. Nine records per job function provide sufficient data to determine inter-rater reliability without placing an undue time burden on the coders. The Experiment 2 dataset was comprised of 49% women, 26% men, and 25% unknown. Figure 3 shows the distribution of records according to year of graduation. For a detailed description of how alumni records were selected for Experiment 2, please refer to *Extended data:* S5.

### Experiment 1

Coders were each provided with three documents: 1) the Draft Guidance Document for how to code challenging records ( *generated by CAS and TD, and piloted at Emory prior to this study;* included within *Extended data:* S2 – note that the Exp 1 version is simply the Exp 2 version without the additions annotated in yellow); 2) UCOT Exp1 with definitions (see *Extended data*: S2 minus the annotated additions); and 3) a Data Collection Workbook (entry-validated spreadsheet designed to collect the data; see *Extended data:* S7) containing the 572 records to be coded using data-validated fields for classification in each tier. Each record was composed of a unique record number, job title, current employer, LinkedIn profile or other job-related URL, and graduation date. Each record in the workbook was coded by selecting from drop-down menus of categories in each taxonomic tier (Workforce Sector, Career Type, and Job Function). For each record, coders were also prompted to indicate (yes/no) whether they had accessed the provided LinkedIn profile. In order to combine the data, since each column/variable used identical headers to indicate which sector was being coded, a rater number was added to each to identify which source worksheet it had originated (e.g., Sector 1_Rater 1, Type_Rater 1, Function_Rater 1; Sector 1_Rater 2, and so forth). Data was collected in separate MS Excel spreadsheets and uploaded into the SPSS software package (Version 24) in preparation for data analysis.

To examine categorical consistency across coders, we used Krippendorff's Alpha ^26^, which estimates the level of agreement ( *inter-rater reliability*) among coders. Parameters of acceptable reliability using Krippendorff's alpha start at a lower bound of 0.67 ( *for a review, see* 27 *; estimates range from 0.67 – 0.80*), with 0.70 being the convergent recommendation used to define reliability among coders ( 25 *gives the caveat that 0.67–0.80 should be interpreted with caution*). Hence, we set 0.70 as our comparison point for an acceptable measure of reliability and replicability of the taxonomies tested in this work. Inter-rater reliability was calculated using SPSS, with an amended macro designed for Krippendorff's alpha ^28^.

In addition to calculating inter-rater reliability, we employed two methods -- average number of unique categories and percent discordance -- to identify discordance between coders. Both methods share a common starting point, which was to determine the Workforce Sector, Career Type, and Job Function that was most commonly chosen (the mode) by the coders for every record. The mode was then designated as the presumed “correct” answer. To calculate the average number of unique categories per tier for each record ( *columns A and C in* *Extended data:* S6), we counted the number of unique categories that coders applied to the given record. We then calculated the average number of unique categories across all records in each tier (as defined by the most popular choice). Percent discordance was measured by determining, for every record, how many coders did *not* choose the same category as the mode. Subsequently, that number was divided by the total number of coders and multiplied by 100 to give a percentage ( *columns B and D of* *Extended data:* S6).

### Revision of the Guidance Document and creation of UCOT Exp2

Following Experiment 1, the group of six coders met twice by phone to review the data, discuss sources of discordance (see Figure 1 and Methods), and ways to improve consistency between coders. These meetings resulted in UCOT Exp2, which was tested in Experiment 2, and a new version of the Guidance Document (“Revised Guidance Document”) that was significantly expanded and refined from the draft version used in Experiment 1. Modifications within the Revised Guidance Document included: a) a formal introduction to the career taxonomy classification system and recommendations for implementation; b) a set of frequently asked questions (FAQs); c) the taxonomic categories with refined definitions; and d) a reference table containing the list of Carnegie Classifications of Academic Institutions to aid in the classification of Career Types as “Primarily Research” or “Primarily Teaching”, if needed (see Discussion). The guidance FAQs addressed and provided proscriptive clarification for how to code types of jobs that had been identified as problematic in Experiment 1, as well as explanations for the logic behind the recommendations. Examples included how to:

- Code training positions ( *e.g., Fellows, Scholars, Residents, Interns*)
- Distinguish between “Principal Investigators” and “Research Staff”
- Define “Entrepreneur”
- Assign a primary job function for individuals with multiple roles
- Differentiate “For-Profit” from “Nonprofit” entities
- Differentiate “Academic” from “Nonprofit” hospitals
- Resolve assignment of faculty members to Career Type “Primarily Research” or “Primarily Teaching”
- Implement the Faculty Flag system to further classify alumni in faculty roles (see Discussion).

### Experiment 2

A new set of 219 alumni records was selected and coded using UCOT Exp2 and the Revised Guidance Document (see annotated changes in *Extended data:* S2). The coding team included five experienced coders from Experiment 1 and three inexperienced coders who were new to the project. New coders were intentionally recruited to participate in Experiment 2 to explore whether the Revised Guidance Document and definitions of updated taxonomic categories were sufficient to increase the reliability of coding by an inexperienced user.

In Experiment 2, coders were provided with: 1) the Revised Guidance Document; 2) UCOT Exp2 ( *Extended data:* S1); and 3) a fresh Data Collection Workbook containing 219 alumni records to be coded using data-validated fields for classification in each tier. The Data Collection Workbook used in Experiment 2 included the Carnegie Classification of each academic employer ^29^ and three new open-answer coding fields, including: 1) drop-down menu related to collecting Faculty Flag data ( *if relevant, as discussed below*); 2) prompt to note whether additional sources had been accessed ( *e.g., institutional website, personal lab website, etc.,*); 3) prompt to indicate how much time was spent coding the record in minutes.

Upon completion of the coding phase of Experiment 2, inter-rater reliability analyses were performed on the results and the group of eight coders met by phone to identify where ambiguity remained in UCOT Exp2. Two areas of confusion were discussed (specifically, classifying the Workforce Sector of K-12 school teachers and the Job Function of entrepreneurs) and the Revised Guidance Document was modified (creating the Final Guidance Document) to provide more detail on how to code alumni in these career paths; no changes were made to UCOT Exp2. *Extended data:* S1 and S3 contain the UCOT Exp2 and the Final Guidance Document, respectively.

We provide summaries of the tiers, categories, and definitions used in Experiment 2 in Table 1– Table 3.

**Table 1.** UCOT Exp2 - Tier 1 Workforce Sector. An individual’s Workforce Sector generally reflects the type of company or institution where they are employed. The unabridged version of UCOT Exp2 including example titles is available in *Extended data:* S1.

WORKFORCE SECTOR
**Academia**
**Government**
**For-Profit**
**Nonprofit**
**Other**
**Unknown**

**Table 2.** UCOT Exp2 - Tier 2 Career Type. An individual’s Career Type should reflect the general content of their work. The unabridged version of UCOT Exp2 including example titles is available in *Extended data:* S1.

CAREER TYPE	Definition	Coding clarifications and examples
**Primarily Research**	The primary, although not necessarily only, focus is the conduct or oversight of scientific research.	See guidance document for suggestions on how to code faculty titles. *Includes postdoctoral research positions.
**Primarily Teaching**	The primary, although not necessarily only, focus is education and teaching.	See guidance document for suggestions on how to code faculty titles. *Includes postdoctoral teaching positions.
**Science-Related**	Career that is relevant to the conduct of scientific research, but does not *directly* conduct or oversee research activities.	Program Officer; Physician; Medical Science Liaison; Editor; Healthcare Consultant
**Not Related to Science**	Career that is not directly relevant to the conduct of scientific research.	Bank Manager; Campaign Manager; Golf Instructor; Chef; Painter
**Further Training or** **Education**	Temporary training position or enrollment in further education.	Law School Student; MD/PhD Student; AAAS Fellow
**Unemployed/ Unknown**	Not currently employed or no information is known.	Family caretaker; retired; unemployed.

**Table 3.** UCOT Exp2 - Tier 3 Job Function. An individual’s Job Function is defined by specific skill sets and/or credentials required for employment in that function. The unabridged version of UCOT Exp2 ( *Extended data:* S1) includes Job Function, Definition, Coding Clarifications, Job Title Examples, and Common Coding Scheme.

JOB FUNCTION	Definition	Coding clarifications
**Administration**	Administrative-intensive roles in which the individual is managing people, projects, and resources.	Spends more than 50% of their time in administrative duties that are internal to the organization.
**Business Development,** **Consulting, and Strategic Alliances**	Role relevant to the development, execution, management, or analysis of a business. Role may include external relationship management, refinement of operational efficiency, or fee-based advisory services.	Director or higher-level positions that have primarily *externally-facing* responsibilities
**Clinical Research Management**	Role that is responsible for the oversight, management, or design of clinical research trials.	
**Clinical Services**	Role that involves that administration of clinical services or research.	
**Data Science, Analytics, and** **Software Engineering**	Role that primarily involves programming, analytics, advanced statistics, data communication, and/or software development.	
**Entrepreneurship**	Founder or co-founder of their own business or enterprise, and employer of at least one other person.	This category should not be used for C-suite executives of established companies, they should be classified as “Business Development” unless they were also involved in founding the company. Freelance specialists should be classified in the field of their specialization ( *e.g., science* *communications, consulting*).
**Group Leader or Principal** **Investigator**	Role that involves leading a research team in any research environment. The individual is clearly responsible for funding and managing a research group.	This job function will be used in multiple sectors (academia, nonprofit, for-profit, government).
**Healthcare Provider**	Role where the primary responsibility is providing healthcare.	These individuals usually fall into the Career Type “Science-related,” unless it is clear that they are primarily performing research; in that case, the career type might be “Primarily Research.”
**Intellectual Property and Law**	Role that involves the curation, management, implementation or protection of intelligence and creation, including patents, trademarks, copyrights, or trade secrets.	Law students belong to this Job Function (instead of “Completing further education”) because their career outcome is specific. Their training status will be captured at the Career Type level as “Further Training.”
**Postdoctoral**	A temporary mentored training position following completion of doctoral degree.	At the Career Type level, classify the postdoc according to the type of postdoctoral research that they are engaged in, likely either “Primarily Research” or “Primarily Teaching.”
**Regulatory Affairs**	Role that involves controlling or evaluating the safety and efficacy of products in areas including pharmaceuticals, medicines, and devices.	
**Research Staff or Technical** **Director**	Role that directly involves performing and managing research.	The individual is involved in research, but not responsible for funding and managing a group. “Staff fellow” is coded here if it is not an official postdoctoral position.
**Sales and Marketing**	Role that is related to the sales or marketing of a science-related product or service.	A medical science liaison should be classified as “Technical Support and Product Development.”
**Science Education and** **Outreach**	Role that involves K-12 teaching or public outreach at a primary/secondary school, science museum, scientific society, or similar.	A public school belongs in the Government Sector.
**Science Policy and Government** **Affairs**	Role that involves policy or program development and review, including analysis, advisory, or advocacy.	Unless a program officer is actively involved in determination of policy, that individual should be classified as “Administration.”
**Science Writing and** **Communication**	Role that involves the communication of science-related topics.	
**Teaching Faculty or Staff**	Teaching position at post-secondary schools, universities, or institutions with no contractual research responsibilities.	Positions in elementary and high schools should be coded as “Science Education and Outreach.”
**Technical Support and Product** **Development**	Role that requires specialized technical/ expert knowledge of a science-related product.	
**Other Employment**	Role that does not require scientific training or involve the direct implementation or communication of science.	Includes: Industry not related to science; politics not focused on science; food or hospitality services; military service; volunteer/mission work.
**Completing Further Education**	Pursuing additional education that usually results in graduation with conferment of a degree or certificate; this does not include traditional academic postdoctoral positions which have their own Job Function “Postdoctoral.”	Pursuing additional education where the career outcome is not specific or clear. Postdoctoral trainees should be classified in the "Postdoctoral" Job Function and Career Type based on their broad field.
**Unemployed or Seeking** **Employment**	Known to be temporarily out of the workforce but likely to return.	
**Deceased/ Retired**	Permanently out of the workforce.	
**Unknown**	No title or career information can be found for this individual.	

## Results

In this study, we tested the consistency and applicability of the Unified Career Outcomes Taxonomy (UCOT 2017; ^23^) and a guidance document that we generated for this study. This was done by assessing the inter-rater reliability of classifications generated by multiple coders using the career taxonomy. Inter-rater reliability was evaluated using three measures: Krippendorff’s Alpha, number of unique category classifications, and percent discordance. We repeated the experiment with iterative refinements to the career taxonomy and guidance document until differences across coders were sufficiently reliable based on published thresholds for Krippendorff’s Alpha.

**Figure 4.**
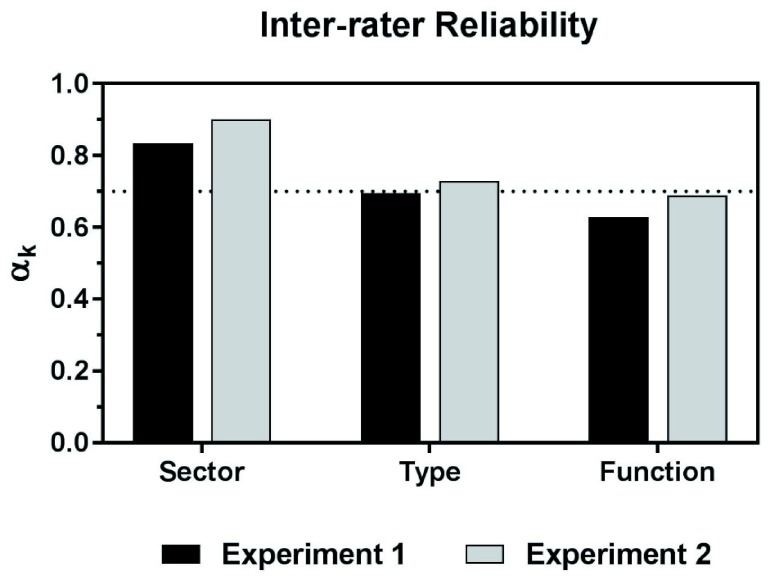
Inter-rater reliability scores of coding for Experiment 1 and Experiment 2, as measured by Krippendorff's alpha (α _k_). Workforce Sector, Career Type, and Job Function were coded by six coders in Experiment 1 (black bars) and eight coders in Experiment 2 (gray bars). The 0.7 threshold is indicated by the horizontal dotted line. Experiment 2 results for every category are higher than Experiment 1. Coders used 6 categories for Workforce Sector, 6 categories for Career Type, and 26 (Experiment 1) or 23 (Experiment 2) categories for Job Function.

Based on our preliminary application of UCOT 2017 ( *data not reported here*), we initiated Experiment 1 by modifying UCOT 2017 to clarify several of the definitions of taxonomic categories, therein generating UCOT Exp1. This version was used to code 572 records. Using a Krippendorff’s Alpha of 0.70 as the minimal threshold to define reliability among six coders (see Methods; ^25, 26^), we observed that the Workforce Sector tier (0.83 agreement) and the Career Type tier (0.70 agreement) met acceptable parameters of reliability ( Figure 4). However, the measured reliability for the Job Functions tier (0.62 agreement) was below admissible levels ( Figure 4).

The six coders in Experiment 1 had six-way agreement on 77% of all records within Workforce Sector, 55% of all records within Career Type, and 36% of all records within Job Function. Furthermore, we reached five-way agreement on 95% of records for Workforce Sector, 95% of records for Career Type, and 73% of records for Job Function, suggesting that, in many cases, only one person interpreted a definition differently than the rest of the group.

To identify hot spots of discordance, we ranked the records by number of unique categories utilized by the coders and looked for patterns among records that had high disagreement (an illustration of the discordance is provided in *Extended data:* S6). Within the Career Type tier, we identified discordance in three of the six categories, specifically, application of the “Further Training” category and in differentiating “Primarily Research” and “Primarily Teaching” faculty positions. Within the Job Function tier, we discovered that seven of twenty-three categories presented high levels of discordance, including: “Faculty- Non-Tenure track”; “Clinical Services”; “Sales and Marketing”; “Group Leader”; “Business Development, Consulting, and Strategic Alliances”; “Clinical Research Management”; and “Administration”.

While we hypothesized that most instances of discordance could be addressed by providing additional examples in the Draft Guidance Document or by making minor clarifications in taxonomic definitions, we also hypothesized that two structural changes to UCOT Exp1 would improve reliability. To address the discordance in the coding of the five faculty Job Functions in UCOT Exp1 (“Adjunct/part-time teaching staff”, “Faculty: non-tenure track”, “Faculty: tenured/tenure track”, “Faculty: tenure track unclear or not applicable”, and “Instructor/full-time teaching staff”), the group agreed to test an alternative approach to capturing faculty data, originally developed by the Biomedical Research Education and Training office (BRET) at Vanderbilt ( *KP and AB, unpublished study*). Specifically, in addition to classifying each record by the three primary taxonomic tiers, the record is also examined for any indication in the job title that the individual holds a faculty designation at their employing institution. If so, the record is ‘flagged’ as faculty and additional information is collected, including title ( *e.g., professor, instructor, lecturer*), rank ( *e.g., assistant, associate*), and prefix ( *e.g., adjunct, research, teaching*). This strategy for capturing faculty status permitted us to combine the five faculty-related Job Functions in UCOT 2017 and UCOT Exp1 into just two Job Functions in UCOT Exp2: “Principal Investigator or Group Leader” and “Teaching Faculty or Staff.” Examples of the Faculty Flag schema with sample data are provided in the Final Guidance Document ( *Extended data:* S3). The second structural change was introduced into the Career Type tier by adding an “Other” category; this permitted coders to more accurately capture alumni for whom some information is known, thereby obviating the default classification as “Unknown”. Incorporation of these changes resulted in the UCOT Exp2, which was then used in Experiment 2 by a second group of coders to code a new dataset of 219 records (see Methods). Among the eight coders for UCOT Exp2 we calculated Krippendorf’s alpha for the Workforce Sector tier (0.90 agreement), the Career Type tier (0.73), and the Job Functions tier (0.69 agreement). Notably, we observed improved inter-rater reliability across all three tiers in Experiment 2 ( Figure 4).

**Figure 5.**
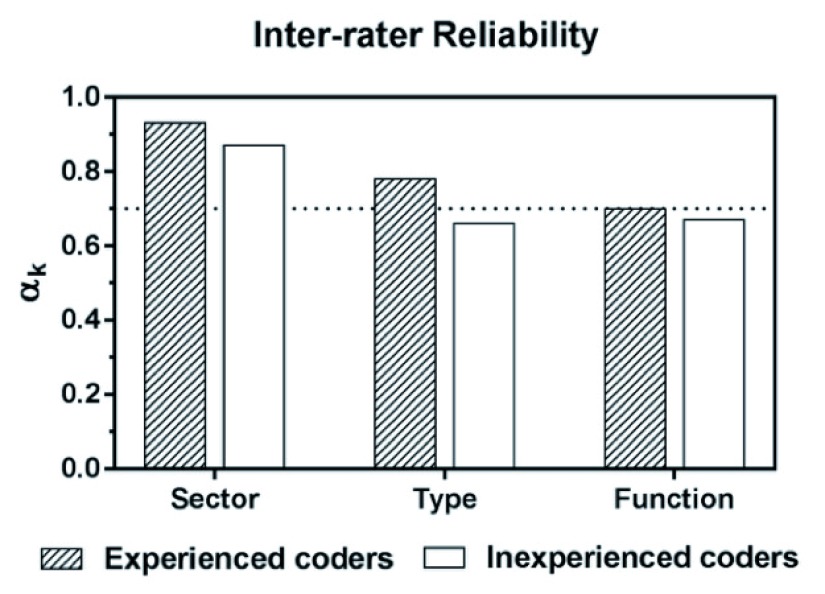
Experiment 2: Inter-rater reliability scores comparing experienced and inexperienced coders, as measured by Krippendorff's alpha (α _k_). In Experiment 2, which included experienced and inexperienced coders, the results are similar for experienced (n=5, striped bars) and inexperienced coders (n=3, white bars). Of note, experienced coders met acceptable standards of reliability for each tier. The 0.7 threshold is indicated by the horizontal dotted line.

We considered that the elevated reliability achieved in Experiment 2 using UCOT Exp2 may have resulted from the coders’ greater familiarity with the career taxonomy, developed through Experiment 1 and subsequent conversations about sources of discordance. To explore this possibility, reliability was calculated separately for experienced and inexperienced coders in Experiment 2. Although experienced coders performed slightly better, inexperienced coders did not lag far behind ( Figure 5). Experienced coders using UCOT Exp2 met standards for acceptable reliability for each tier, including Krippendorf’s alpha for the Workforce Sector tier (0.93 agreement), the Career Type tier (0.78), and the Job Functions tier (0.70 agreement).

The introduction of inexperienced coders to Experiment 2 also permitted us to explore whether the amount of time spent coding each record significantly differed between inexperienced and experienced coders. Accordingly, in Experiment 2 all coders were prompted to record how much time was spent coding each record. The overall average time to classify the second round of 219 records was 366 minutes, which equates to about 1 minute, 40 seconds per record.

The total time for experienced coders ranged from 213 to 540 minutes, on average 328 minutes, or about a minute and a half. In comparison, the total time for inexperienced coders ranged from 246 to 545 minutes coders, on average 429 minutes, or about two minutes. While this may not seem like a huge difference in time to code records for inexperienced verus experienced coders, depending on the size of one’s alumni populations, this may affect one’s approach. For instance, if binning 1000 records, taking the time to become a trained “experienced coder” would save each coder approximately a day’s worth of full-time effort (e.g., approximately 33 hours versus 25 hours).

### Description of changes made to UCOT 2017 to increase reliability

In the process of identifying discordances through this study, we adapted the UCOT classification system and refined our corresponding guidance document to improve reliability. These changes are detailed below. See also *Extended data:* S2, which contains annotations for each change comparing UCOT Exp2 with the original UCOT 2017 (this also allows for ease of comparison and/or cross-walking one’s data between versions as needed).

***Tier 1: Workforce Sector.*** In the Workforce Sector tier, we defined Academia as “any post-secondary academic institution where training occurs, including colleges, universities, some medical centers, or freestanding research institutions.” We found discordance among coders when assigning K-12 teaching jobs to Workforce Sector and, as a result, we concluded that K-12 schools would be coded as Government, Nonprofit, or For-Profit based on publicly-available information about that school. For example, public K-12 schools are most properly classified in the Government sector, and private schools are either For-Profit or Nonprofit (see *Extended data:* S3).

***Tier 2: Career Type.*** In the Career Type tier of UCOT Exp2 we added the term ‘unemployed’ to the existing category “Unknown,” renaming this category “Unemployed/Unknown.” In the absence of this category, individuals who are not currently employed but who are known to be searching for a job or intend to return to the workforce could only be classified as Career Type “Unknown,” thereby losing information that may actually exist about their current employment status. Now, by classifying them as “Unemployed/Unknown” at the Career Type tier and combining that with a new Job Function category, “Unemployed or Seeking Employment” ( *described below*), we are able to capture current status of individuals who have described themselves as, for example, family caregivers or on the job market.

Second, classification of faculty as “Primarily Research” versus “Primarily Teaching” is complicated by the multi-functional roles played by many faculty members and by the fact that a large proportion of academic faculty balance both research and teaching. We instructed the coders to use available public resources to determine in which capacity the faculty member is likely to spend most of their time. Examples of this type of data included but are not limited to: frequency and recency of peer-reviewed research articles (e.g., publication record on PubMed); information related to the number of courses that an individual taught in a given academic year; and, supervising the research of doctoral students or postdoctoral scholars.

If no illuminating data could be found to facilitate classification of a given faculty member as “Primarily Research” or “Primarily Teaching”, we stipulated that a coder should use the Carnegie Classification ^29^ of the employing institution to classify Career Type. For example, if the individual was a faculty member at an academic institution with a Carnegie Classification of 15 or 25 (“highest research activity”), the coder should categorize the individual as “Primarily Research”. Faculty at institutions in any other Carnegie Classification should be categorized as “Primarily Teaching.” (See further examples of this coding strategy in the Final Guidance Document, *Extended data:* S3). We urged coders to gather data from online sources listed above and to avoid using the Carnegie Classification strategy due to its potential inaccuracy. For example, defaulting to the Carnegie Classification would misrepresent some faculty as “Primarily Research” who are actually “Primarily Teaching” because many institutions with Carnegie Classification 15 or 25 hire faculty members to serve in primarily teaching roles such as instructors or teaching professors. This default strategy could also underreport the research responsibilities held by many faculty members at institutions with Carnegie Classification other than 15 or 25.

Third, we addressed the classification of postdoctoral scientists in the Career Type tier. In UCOT 2017, postdoctoral scientists are classified as “Further Training or Education” at the Career Type tier, followed by “Postdoctoral” at the Job Function tier, therein losing the specific nature of their postdoctoral work. For example, if the individual is engaged in a postdoctoral training program with a significant teaching focus, the UCOT 2017 taxonomy does not capture the teaching emphasis of their training. As a consequence, one could lose opportunities to uncover distinct shifts among PhD outcomes, such as identifying longitudinal trends in the types of postdoctoral training experiences pursued by our trainees (e.g., a decrease in number of research-focused postdocs accompanied by a rise in teaching-focused or government policy-focused postdoctoral positions). Facilitated by the possibility that their postdoctoral training status could be captured in the Job Function tier, we instructed coders to classify postdocs as “Primarily Research” or “Primarily Teaching” instead of as “Further Training or Education” ^6, 30, 31^. This strategy allowed us to collect additional detail of the *nature* of their postdoctoral experience and greater resolution pertaining to a population that the academic community would like to track and analyze more precisely. By using the more detailed classification during data collection and coding, it is easy to later choose to report postdocs consistently with UCOT 2017 or UCOT Exp2, as desired. Alternative methods which would satisfy both reporting options are discussed further (below).

Some institutions using UCOT 2017 are collecting and reporting data for tiers 1 and 2 (Workforce Sector and Career Type), but not for tier 3 (Job Function). This raises a caveat to our proposed solution for categorizing postdoctoral scientists: in the instance where an institution is not collecting the Job Function tier, we agree that postdocs should be classified as “Further Training or Education” in Career Type (tier 2) to highlight the temporary nature of the postdoctoral position. Otherwise, the institution will undercount the number of graduates going on to postdoctoral positions.

Our proposed solution is one of many potential solutions to this challenge of collecting more nuanced detail about postdoctoral outcomes. Alternate strategies could include:

- creating additional Job Functions to capture different types of postdoctoral positions (e.g., “Postdoctoral: Research” and “Postdoctoral: Teaching”);
- implementing a “Postdoc Flag” to capture the focus of the postdoctoral position (e.g., research, teaching, policy, etc.). In the latter case, the individual would be classified as Career Type “Further Education or Training” (as is currently recommended in UCOT 2017), followed by Job Function “Postdoctoral,” and an additional data collection field would prompt the coder to select the focus of the postdoctoral position (research, teaching, policy, etc.); or,
- removing postdoctoral/further education/training categories from Career Type and Job Function tiers, then introduce a “Training Flag” (denoting “Student” or “Postdoctoral”) in conjunction with classifying them in a category that is most similar to their type of work.

Regardless of strategy, we recommend erring on the side of collecting as much data as possible from the beginning of the process, as it is more efficient (e.g., less time- and resource-intensive) to collect data points all at once than it is to go back to gather additional data as an afterthought.

As a corollary, it may be appropriate for the “Further Training or Education” category within the Career Type tier to be reserved explicitly for specialized training experiences *distinct from the traditional research or teaching postdoc*; examples include science policy fellowships ( *e.g., American Association for the Advancement of Science (AAAS) Science and Technology Policy Fellowship*) or academic programs that will lead to an additional degree or certification ( *e.g., law school, MD/PhD students in second stage of training*). When this strategy is used, we suggest that the focus of the further training can be captured at the Job Function tier. For example, a student in law school would be classified as “Academia → Further Training or Education → Intellectual Property and Law.” Similarly, an AAAS Fellow would be classified as “Nonprofit → Further Training or Education → Science Policy and Government.” The Final Guidance Document ( *Extended data:* S3) includes additional examples of how an institution might choose to apply Job Function categories to postgraduate and postdoctoral training programs.

***Tier 3: Job Function.*** Through iterative experimental modifications, we identified three clarifications to definitions in the Job Function tier and one larger structural modification which increased concordance among coders. Below we summarize these modifications; detailed definitions and annotated changes are provided in *Extended data:* S2.

First, we added a new Job Function category “Unemployed or Seeking Employment” to more accurately classify individuals for whom some information is known about their employment status ( *e.g., individuals who are known to be family care providers or seeking employment).* In UCOT 2017, all of these individuals would be classified as Job Function “Unknown.” Capturing data about these individuals will help institutions track the individuals who are intentionally not employed (family care providers) and those who are actively looking for employment.

Second, we found that our group faced significant challenge in distinguishing between the Job Functions “Administration” and “Business Development, Consulting, and Strategic Alliances”. Upon further discussion, we concluded that discordance resulted from specific job titles such as “Chief Financial Officer” or “Academic Dean” that relate to the administration of a company/institution (and therefore might be classified as “Administration”) yet entail significantly more responsibility and a different skill set than a departmental administrator or program manager. We suggest that these Job Functions could be differentiated by the *primary focus of their work* (defined as 50% or more of an individual’s time spent on these duties): strictly administrative roles spend most of their time in duties that are *internal to the organization*, while business development roles are generally higher-level positions that have *externally-facing responsibilities*.

Under this revised definition, a dean of the biological sciences division would be classified as “Academia → Science-Related → Business Development, Consulting, and Strategic Alliances,” while a departmental administrator would likely be classified as “Academia → Science-Related → Administration.”

Extrapolating to other Workforce Sectors, a project review manager at a For-Profit industry company would be classified as “For-Profit → Science-Related → Administration,” while the director of medical affairs at the same for-profit industry research company would be classified as “For-Profit → Science-Related → Business Development, Consulting, and Strategic Alliances.” In the Government Sector, a grant administrator at National Institutes of Health would likely be classified as “Government → Science-Related → Administration,” and the director of the National Institutes of Health would be classified as “Government → Science-Related → Business Development, Consulting, and Strategic Alliances.” We recognize that the individual tasked with classifying alumni records may not be able to distinguish the full spectrum of duties involved in a specific job and may feel ill-equipped to categorize these individuals. We encourage coders to use public data sources to their fullest extent, accepting that, as in all projects that are based in subjective assessment, there will be some degree of error introduced by interpretation.

Third, we identified discordance generated by job titles that included ‘medical science liaison’, which UCOT 2017 classifies as a “Sales and Marketing” Job Function. We discovered that, even within our group of career development professionals, there were different understandings of what a medical science liaison actually does on a day-to-day basis; this is an example of a job that has evolved significantly over the past decade and can vary greatly depending on the employer. After significant discussion and outreach to individuals who hold the ‘medical science liaison’ job title, we concluded that, by default, these individuals should be classified in the “Technical Support and Product Development” Job Function. We recognize that there may be exceptions to this rule and encourage coders to obtain as much publicly-available information as possible about the individual’s actual duties to make the most informed categorization for each unique individual.

A significant modification we made that dramatically increased inter-rater reliability and the ease of coding faculty members relates to the classification of academic faculty and teaching staff. In UCOT 2017, academic faculty are classified according to their tenure status: “Faculty: Tenure track”, “Faculty: Non-tenure track”, or “Faculty: Track unclear or not applicable.” During preliminary use of UCOT 2017 ( *not reported here*), multiple classification strategies emerged among the coders. Some coders assumed tenure status based on the institution’s Carnegie Classification (R1, R2, etc.) and, in the absence of an ‘adjunct’ preface in their faculty title, classified nearly all faculty positions as “Faculty: Tenure track.” Other coders concluded that a tenure-track assignment could not be made without direct confirmation from the individual, given that many institutions appoint non-tenure track assistant professors or have eliminated the traditional tenure structure altogether ^19^. We anticipated that our larger-scale experimental application of UCOT 2017 would reveal the same inconsistencies in application of these categories and thus, in the Draft Guidance Document provided to coders prior to initiating Experiment 1 ( *see* Figure 1 *for experimental procedure*), we included the specific instruction *not* to code faculty as “Faculty: Tenure-track” *unless* the coder was absolutely certain that the individual held a traditional tenure track faculty appointment. This clarification of instruction resulted in zero alumni records being classified as “Faculty: Tenure-track” category during Experiment 1 ( *Extended data:* S6). Even so, we *still* observed high discordance in application of the other faculty-related Job Functions (“Faculty: Non-tenure track” and “Faculty: Track unclear or not applicable”) and the determination of Career Type as “Primarily Research” or “Primarily Teaching.”

In light of the inability of multiple career development professionals to consistently and accurately assess the tenure status of an individual based on their job title and home institution, as well as changing attitudes about academic tenure ^32^, we suggest that individuals with academic titles, such as professor, instructor, adjunct professor, research associate professor, be classified under “Principal Investigator or Group Leader”, “Research Staff or Technical Director”, or “Teaching Faculty or Staff”, based on information sourced from an individual’s public web presence ( *PubMed, LinkedIn, institutional or personal web pages*). In order to permit finer resolution of the faculty population, we recommend that each faculty record be identified by a Faculty Flag and further notated with their faculty title, rank, and prefix (see Methods and *Extended data:* S3). In our experiment, adding the Faculty Flag to UCOT Exp2 greatly reduced discordance in the classification of faculty positions ( *Extended data:* S6).

Implementation of the Faculty Flag in UCOT Exp2 yielded multiple benefits. Five previous faculty Job Functions (“Adjunct/part-time teaching staff”, “Faculty: non-tenure track”, “Faculty: tenured/tenure track”, “Faculty: tenure track unclear or not applicable”, and “Instructor/full-time teaching staff”) were reduced to two (“Principal Investigator” and “Teaching Faculty or Staff”), resulting in a more simplified career taxonomy. Furthermore, our coders captured and categorized *more information* from alumni records than just a presumed tenure status. Specifically, this strategy allowed coders to use the Job Function category to describe the functional duties that comprised the majority of a faculty member’s time, without undercounting or misreporting their faculty appointment. For example, the Faculty Flag permitted coders to classify faculty who hold multiple academic titles, such as deans and directors, as having a “Business Development, Consulting, and Strategic Alliances” (e.g., Provost, University Chancellor/President) or “Administrative” (e.g., Chair, Dean) Job Function, respectively, while simultaneously recording their faculty status. This is intended to enhance the career taxonomy’s ability to identify faculty with broad leadership roles (externally or internally, respectively), rather than failing to recognize the impactful nature of their work within the academy and beyond. We acknowledge applying the category “Business Development, Consulting, and Strategic Alliances” to faculty and other institutional leaders may be confusing at first glance. Hence future revisions may consider using the same words reordered to lead with Strategic Alliances (e.g., “Strategic Alliances, Business Development, and Consulting”), since this function may be more readily recognizable in reflecting individuals who hold multiple roles. For additional examples of the versatility of the Faculty Flag approach, please see *Extended data:* S3.

Finally, removing the focus on tenure from the faculty Job Function categories allowed us to identify similar roles across Workforce Sectors. Specifically, the definition “a role that involves leading a research team in any research environment where the individual is clearly responsible for funding and managing a research group” equally describes a principal investigator at an academic institution and a group leader at an industry research company. Therefore, we combined the two into a single Job Function category: “Group Leader or Principal Investigator.” This combination can easily be re-sorted into faculty and non-faculty subgroups as needed more detailed demographic analysis, while structurally reflecting the similar roles played in these two roles. To disambiguate the combination, the Workforce Sector classification (based on the individual’s employer) will clarify whether the individual performs this function in an Academic, For-Profit, Nonprofit, or Government environment. By recognizing the homology that exists between principal investigators in academia and group leaders in other sectors, UCOT Exp2 underscores the suitability, relevance, and contributions of PhD-trained scientists across the workforce.

We recognize that the collection and classification of records at the granular resolution of Job Function may seem burdensome or unnecessary to some institutions. Also, while Job Function data is extremely useful in developing career support services internal to the institution, it may be less relevant to national reporting standards. Indeed, the NGLS institutions have agreed to publish the Workforce Sector and Career Type tiers and are not requiring public reporting of the Job Function tier. However, we strongly recommend the collection of Job Function data, even if it is not publicly disseminated, since the data can have great value at the local, intra-institutional, and national levels. More than Work Sector or Career Type, Job Function data provides valuable insight into the types of skills sets used by PhDs and can inform the ongoing development of graduate and postdoctoral training support services to address changing needs in the scientific workforce. Job Function data also serves the current and prospective trainees who can gain insight into the breadth of career paths available to them and then leverage alumni who have pursued specific careers to make more informed career decisions for themselves. PhD training endows individuals with a versatile skill set, as demonstrated by PhDs who become serial entrepreneurs, government policy advisors, science educators, and public communicators, all contributing to society in ways that can be claimed and espoused by the institutions that prepared them to be successful in those ways. Collecting and publicly disseminating the breadth of career paths that PhDs pursue is essential for institutions, policy makers, and the public to assess the impact of their investment in the PhD training enterprise.

## Discussion

In this study, we tested the inter-rater reliability of multiple coders categorizing the records of PhD alumni using a collaboratively-established, three-tier career outcomes taxonomy, UCOT 2017. Through two rounds of refinement (Experiment 1 and Experiment 2) of UCOT 2017 and a companion guidance document, we achieved satisfactory inter-rater reliability across all three tiers of the career taxonomy. We ascribe the majority of the improvement in reliability during Experiment 2 ( Figure 4) to the provision of a detailed Revised Guidance Document, which was largely informed by the classification of 2500+ alumni records prior to the initiation of experiments reported here. For the experiments in this study, we intentionally enriched the alumni data set of 800 records with less common Job Functions to ensure full coverage of the career taxonomy, with the hope that we would encounter uncommon types of employment that were poorly defined or described in the taxonomy. While there are likely to be unique forms of employment that we did not anticipate or encounter in our datasets (and therefore are not addressed in the Final Guidance Document), we are confident that we obtained full coverage of the most common current career outcomes for PhDs in the biosciences and that the Final Guidance Document ( *Extended data:* S3) provides sufficient instruction to reliably code these career outcomes. The strength of UCOT 2017 is evident in its overall performance in our study, given the increased representation of challenging job titles in our alumni samples.

Our experiments indicate that institutions should consider the value of training and retaining experienced coders year after year; however, the use of inexperienced coders should not preclude an institution from starting to classify alumni career outcomes ( Figure 5). As coders are first trained, we recommend that institutions internally measure and evaluate inter-rater reliability on sample records. To that end, institutions or academic divisions may find that it is most efficient to designate a small, centralized team to code all alumni records, as opposed to approaching classification in a more decentralized manner that involves many inexperienced coders. We also recommend that groups aiming to use a career taxonomy to compare across institutions meet to develop a detailed guidance document to ensure common interpretation of categorical definitions.

### Considerations in implementing a unified career outcomes taxonomy

Ideally, adoption of a single career taxonomy across all institutions would enable inter-institutional analyses of alumni career outcomes. However, establishing a static and universally-acceptable career outcomes taxonomy across institutions will be challenging and may not be realistic. Our results underscore that clarifications to UCOT 2017 will be needed as the workforce evolves. Additionally, individual institutions or disciplines may find that some taxonomic categories are not fully applicable in their environment; others may choose to alter definitions or create additional job functions to better suit their needs. For any changes that a group introduces to UCOT 2017, we urge institutions to clearly and prominently annotate these definitional differences when displaying the data such that it will be clear when data are comparable or not, hence avoiding cross-institutional data comparisons that are inappropriate. Furthermore, we encourage the community to come together periodically to discuss challenging cases, consider recommended changes, and test and refine inter-rater reliability for new definitions. This will be necessary if we are to sustain a career outcomes taxonomy that enables merged and comparative analyses across institutions. In order to serve both the purpose of evolving the career taxonomy, while simultaneously maintaining the ability to compare data, our guidance document includes “cross-walk” instructions for institutions to easily use their preferred classification choice, while also reporting national outcomes using consistent definitions ( *e.g., CNGLS current reporting requirements*). This can easily be accomplished using the same data set, and very minimal additions to your data collection system. Several groups are already employing variants of UCOT Exp2 as the basis of research projects, having made local adaptations that serve their unique tracking questions ^17, 18^; *personal communications from S.S. Kleppner, PhD, Associate Dean for Postdoctoral Affairs, Stanford University, on 09/22/2019, W.T. McCormack, Professor, Pathology and Immunology & Laboratory Medicine, University of Florida, 10/22/2019; T.L. Dennis, COM, Assistant Director for Postdoctoral Affairs, J.K. McClarin, Office of Academic Career Development, & D.F. Zellers, PhD, University of Pittsburgh on 10/21/2019*]. We propose that the addition of the flag system in UCOT Exp2 provides an adaptable approach that permits institutions to tailor the career taxonomy in order to accommodate a variety of reporting needs.

## Limitations and Future Directions

A major strength of UCOT 2017 is that it was collaboratively developed by a diverse group of scientists, including academic faculty, institutional leaders, and career development professionals. The diversity of this group and the group’s collective knowledge of job functions across sectors lends itself to the validity of this instrument. However, most of the creators were employed or had strong roots in academia, which likely affected the taxonomy’s design and interpretation. Therefore, we must consider that biases were potentially introduced into the career taxonomy due to the academic background of the taxonomy developers. To ensure inclusive representation from all career fields, we recommend that future discussion of changes to UCOT 2017 include employers in sectors outside the academy

A perceived limitation of UCOT 2017 is its specificity to the biological sciences. Interestingly, early application of UCOT 2017 suggests that it can easily be adapted for other disciplines. For example, cross-disciplinary implementation of UCOT 2017 at Wayne State University identified that, after replacing any occurrence of the word ‘science’ in the career taxonomy with the word ‘discipline,’ the existing Career Types and Job Functions were sufficient to classify career outcomes across disciplines ( *A. Mathur, personal communication on 06/22/2018; ^33^*). In practice, in the Career Type tier, changing the category “Science-Related” to “Discipline-Related” or, in Job Functions, changing the category “Science Communications” to “Discipline-Related Communications” made the career taxonomy sufficient for cross-disciplinary implementation, with the single exception of adding the category “Author” to the Job Function tier. As the number of institutions that have adopted UCOT 2017 increases, we encourage continued collaboration across disciplines to further explore the versatility and applicability of the tool.

One criticism of the current taxonomic nomenclature is the use of “Not Related to Science” and “Science-Related” in the classification of Career Type due to the negative connotation associated with a career that is not directly related to one’s training. Specifically, jobs categorized as “Not Related to Science” may be interpreted as unattractive, unusual, or not requiring or valuing PhD training -- none of which may be true. Negative social consequences of the language used to mark a category as ‘other’ have been broadly explored in studies of gender, race, and even mathematical skill ^34^. This taxonomy’s definition of “Not Related to Science” is “a career that is not directly relevant to the conduct of scientific research”, and, while we may agree that there are job types that fit that definition, one should not assume that the scientific skills and/or knowledge developed during research training are not used in those roles. For example, “children’s book author” may seem to be “not related to science,” however the author may publish books with science themes based in their scientific training. Because detailed information on the nature of work will not be available in all cases, we accept that coders will need to make educated guesses for classification between “Not Related to Science” and other Career Types. We hope that, as scientists innovate new roles for themselves throughout the workforce, the predominant negative valuation of careers ‘not related to science’ will be challenged. We underscore that future iterations of UCOT 2017 could explore better ways to identify and classify the diverse career trajectories of PhD alumni without using “othered” terminology.

It has not escaped our attention that one shortcoming of all versions of UCOT 2017, discussed at length during its generation and refinement over the past three years, is its inability to sufficiently represent situations where an individual balances multiple job functions. For example, there is currently no way to notate the balance of research and teaching that is expected of faculty members who might otherwise be categorized as Career Type “Primarily Teaching” and Job Function “Teaching Faculty or Staff.” Physician scientists and other cross-functional roles provide the same challenge to accurate coding of their position. We anticipate that the ‘flag’ approach could be used in additional ways by institutions that want to collect more nuanced outcome data. For example, one potential solution to this challenge could involve further elaboration of the Faculty Flag fields to include additional ‘yes/no’ drop-down prompts that investigate the contractual expectations of their appointment, e.g., research, teaching, clinical service, and/or service expectation. We recommend that the outcomes tracking community consider whether and how to address this currently unaddressed challenge.

A recognized limitation of our study is that the coding group in our study consisted entirely of career professionals in graduate education; in reality, not all institutions will have a PhD-trained career professional available to do this, nor would the individual in this role necessarily have the time to code and regularly maintain an up-to-date career outcomes database. Consequently, the career taxonomy must be tested by novice coders outside of graduate career and professional development. Initial steps in this direction have been initiated, including at two institutions to date (Stanford University and the University of Chicago). Initial results suggest that, indeed, novices without graduate career development expertise training will need additional training and systems in place to ensure high-quality data coding (e.g., UCSF’s systematic data verification process ^24^; Stanford University’s multi-user reliability check, currently in pilot phase). Further refinement and testing will be needed to ensure reproducible and consistent results across novice coders with different expertise and training backgrounds. In many cases graduate assistants (especially doctoral candidates with an interest in career and professional development or higher education administration careers) could provide a readily accessible solution, leveraging their personal experience in graduate training along with the motivation and interest to learn such taxonomic distinctions. This type of testing and implementation of verification systems will be essential to in the practical application of the career taxonomy as it is deployed nationally.

This iteration of the career taxonomy does not adequately capture the characteristic of self-employment, since self-employed individuals are currently classified by the field in which they are working. When the taxonomy was developed, the original UCOT designers agreed that when a classification could fall within multiple categories in the same tier, it should be considered subsequently in a different tier. In future variations, self-employment could be captured by an additional flag system similar to faculty or postdocs, which may be relevant to institutions and programs with an interest in entrepreneurship startups, and spinoffs.

While this experiment focused on evaluating the consistency and time investment needed for manually classifying career outcomes, future directions to improve efficiency might include development of automated systems or programs to implement career classifications, particularly with the advent of machine learning in the current era. As a first step, perhaps machine-learning could increase efficiency by creating initially proposed binning of career outcomes which could then be spot-checked or confirmed by a human being on a second pass. Perhaps this process could even be fully automated pending technological advances.

## Conclusion

Gathering and reporting longitudinal alumni career outcome data is an important and weighty task, but one that is within reach for all graduate and postdoctoral programs. Data classified via a well-defined career taxonomy can meet the practical needs of graduate students and postdoctoral scholars who are exploring career paths, research institutions that are strategizing for the future of research and education, funding agencies that are investing in the training and growth of the national scientific workforce, and other relevant stakeholders. Having a well-defined and validated career taxonomy to code alumni outcome data will make collecting and understanding career outcomes for PhDs maximally useful internally, cross-institutionally, and nationally. We believe that UCOT 2017, in its original state as made available at the conclusion of the RBR working group, approaches fulfillment of that goal. However, we identified -- first informally via discussion, then rigorously via this study -- that there are several parts of the career taxonomy that permit variable interpretation and that we were unable to apply it reliably. Through an iterative and scientifically rigorous process, we tested whether the career taxonomy met basic standards for inter-rater reliability, and developed an example revision of the taxonomy (UCOT Exp2) which improved inter-rater reliability for all three tiers. We share UCOT Exp2 and the Final Guidance Document to demonstrate that relatively small alterations can improve functional application of the career taxonomy. For those institutions that may be attracted to the additional types of data collected through UCOT Exp2 but want to maintain the option to publicly display data in accordance with UCOT 2017, closer inspection of the differences will reveal that there are simple ways to convert between them. Regardless of taxonomic categories, we encourage institutions to include a clear description of definitions used to classify the records when publishing outcomes data publicly on institutional websites.

Sustaining a unified career taxonomy across many institutions will require working together as a community in an ongoing fashion. Any career taxonomy adopted by an institution or group of institutions will require periodic updating as the career landscape evolves. We recommend that the reliability of a career taxonomy be periodically revisited, tested, and updated accordingly. Testing, maintaining, and evolving any career outcomes taxonomy will benefit from the collective expertise of multiple stakeholders, including PhD career development professionals, employers of PhDs, leaders in graduate and postdoctoral education, funders, and policy makers. As more and more institutions join ranks in providing career and professional development for a wide range of career choices, it makes sense to accurately track and report on the outcomes of alumni along those career paths. It is our hope that the growing momentum on this front will provide enormous benefits to policy makers, employers, training programs, career development professionals, funders, and -- most of all -- current and future trainees.

## Data availability

IRB approval for public data-sharing is limited to de-identified and aggregated data only, due to concerns of sharing personally identifiable information, which could be traced back to identify individuals included in the data set. The personally-identifying job title, employer, and LinkedIn profile or other job-related URL were collected by individual institutions for their own alumni and used for coding purposes. Limited data-sharing for publication was approved by the respective IRBs as noted above, along with internal data-sharing agreements with NIH and the BEST Consortium.

### Extended data

Open Science Framework: Applying inter-rater reliability to improve consistency in classifying PhD career outcomes - Extended Data S1-S11, https://doi.org/10.17605/OSF.IO/5CGUJ ^35^.

This project contains the following extended data:

- S1. Unified Career Outcomes Taxonomy Experimental Version 2 (UCOT Exp2) unabridged
- S2. Annotated table of changes made to variations of UCOT 2017 in generating UCOT Exp2
- S3. Unified Career Outcomes Taxonomy Experimental Version 2 (UCOT Exp2) Final Guidance Document and Crosswalk
- S4. Selection and Composition of Alumni Records for Experiment 1 Data Set
- S5. Selection and Composition of Alumni Records for Experiment 2 Data Set
- S6. Two measures of discordance used to compare coding after Experiments 1 and 2
- S7. Data Collection Workbook template.

Data are available under the terms of the Creative Commons Zero "No rights reserved" data waiver (CC0 1.0 Public domain dedication).

### Underlying data

While it is not possible to share the data that includes the identifying information, de-identified coder-agreement raw data is available alongside the extended data files in the OSF project ( https://doi.org/10.17605/OSF.IO/5CGUJ ^35^):

- S8. De-identified coder agreement data for Experiment 1 Data Set.
- S9. Coding Key for Experiment 1.
- S10. De-identified coder agreement data for Experiment 2 Data Set.
- S11. Coding Key for Experiment 2

Data are available under the terms of the Creative Commons Zero "No rights reserved" data waiver (CC0 1.0 Public domain dedication).

## References

[1] National Institutes of Health: Biomedical Research Workforce Working Group Report. Bethesda: National Institutes of Health.2012 Reference Source

[2] Hitchcock P Mathur A Bennett J: The future of graduate and postdoctoral training in the biosciences. *eLife.* 2017;6: pii: e32715. 10.7554/eLife.32715 29049023 PMC5648525

[3] The future of graduate and postdoctoral training in the biosciences. In: Virginia Commonwealth University Medical School. Richmond, VA. [cited 12 Jul 2018].

[4] Alberts B Kirschner MW Tilghman S: Rescuing US biomedical research from its systemic flaws. *Proc Natl Acad Sci U S A.* 2014;111(16):5773–5777. 10.1073/pnas.1404402111 24733905 PMC4000813

[5] Pickett CL Tilghman S: Becoming more transparent: Collecting and presenting data on biomedical Ph.D. alumni. *PeerJ Preprints.* 2018;6:e3370v2[Preprint] [cited 2018 Jul 12]. 10.7287/peerj.preprints.3370v2

[6] McDowell GS Gunsalus KT MacKellar DC: Shaping the Future of Research: a perspective from junior scientists [version 2; referees: 2 approved]. *F1000Res.* 2015;3:291. 10.12688/f1000research.5878.2 25653845 PMC4304227

[7] Kimble J Bement WM Chang Q: Strategies from UW-Madison for rescuing biomedical research in the US. *eLife.* 2015;4:e09305. 10.7554/eLife.09305 26122792 PMC4484056

[8] Tracking Participation and Evaluating Outcomes. In: NIH BEST Consortium. Nashville: NIH Best Consortium; c2014-2018 [cited 12 Jul 2018]. Reference Source

[9] Mathur A Brandt P Chalkley R: Evolution of a Functional Taxonomy of Career Pathways for Biomedical Trainees. *J Clin Transl Sci.* 2018;2(2):63–5. 10.1017/cts.2018.22 30364657 PMC6197487

[10] Blank R Daniels RJ Gilliland G: A new data effort to inform career choices in biomedicine. *Science.* 2017;358(6369):1388–1389. 10.1126/science.aar4638 29242335

[11] Understanding PhD Career Pathways for Program Improvement. In: Council of Graduate Schools. Washington, DC.2015[cited 12 Jul 2018]. Reference Source

[12] Statement by AAU Chief Academic Officers on Doctoral Education Data Transparency. In: Association of American Universities. Washington, DC.2017[cited 12 Jul 2018]. Reference Source

[13] Institutional Approaches to Tracking Research Trainee Information. In: Association of American Medical Colleges. Washington, DC.2015[cited 12 Jul 2018]. Reference Source

[14] National Academy of Sciences, Engineering, and Medicine: The Postdoctoral Experience Revisited. Washington, DC: The National Academies Press;2014 10.17226/18982 25590106

[15] National Academy of Sciences, Engineering, and Medicine: Graduate STEM Education for the 21st Century. Washington, DC: The National Academies Press;2018 10.17226/25038

[16] Coalition for Next Generation Life Sciences. In: Coalition for Next Generation Life Sciences. 2018 [cited 13 Jul 2018]. Reference Source

[17] Mathur A Cano A Kohl M: Visualization of gender, race, citizenship and academic performance in association with career outcomes of 15-year biomedical doctoral alumni at a public research university. *PLoS One.* 2018;13(5):e0197473. 10.1371/journal.pone.0197473 29771987 PMC5957427

[18] Xu H Gilliam RST Peddada SD: Visualizing detailed postdoctoral employment trends using a new career outcome taxonomy. *Nat Biotechnol.* 2018;36(2):197. 10.1038/nbt.4059 29334368 PMC5872819

[19] Silva EA Des Jarlais C Lindstaedt B: Tracking Career Outcomes for Postdoctoral Scholars: A Call to Action. *PLoS Biol.* 2016;14(5):e1002458. 10.1371/journal.pbio.1002458 27152650 PMC4859534

[20] Fuhrmann CN Hobin JA Lindstaedt B: myIDP, an online interactive career planning tool. AAAS (American Association for the Advancement of Science).Website launched Sept. 7, 2012. Reference Source

[21] Stayart CA Hutto T Baas T: Constructing a Taxonomy for Career Outcome Reporting. In iCollaborative. 2017[cited 12 Jul 2018]. Reference Source

[22] Stayart CA Mathur C Chalkley R: Report from meeting on career outcomes. In: NIH BEST.2017[cited 12 Jul 2018]. Reference Source

[23] Pickett C: Improving transparency in PhD career outcomes. In: Rescuing Biomedical Research.2017[Cited 12 July 2018]. Reference Source

[24] Silva EA Meja AB Watkins ES: Where do our graduates go? A toolkit for retrospective and ongoing career outcomes data collection for biomedical PhD students and postdoctoral scholars. *bioRxiv.* 2019 10.1101/539031 PMC8727058 31702952

[25] Krippendorff K: Content Analysis: An introduction to its methodology.3rd ed. Everly Hills, CA: Sage.2013 Reference Source

[26] Hallgren KA: Computing Inter-Rater Reliability for Observational Data: An Overview and Tutorial. *Tutor Quant Methods Psychol.* 2012;8(1):23–34. 10.20982/tqmp.08.1.p023 22833776 PMC3402032

[27] Taylor J Watkinson D: Indexing reliability for condition survey data. *The Conservator.* 2007;30(1):49–62. 10.1080/01410096.2007.9995223

[28] Hayes AF Krippendorff K: Answering the call for a standard reliability measure for coding data. *Communication Methods and Measures.* 2007;1(1):77–89. 10.1080/19312450709336664

[29] Carnegie Classification of Institutions of Higher Education 2015 Update Public File. In: Indiana University Center for Postsecondary Research. Bloomington, IN.2015[cited 13 Jul 2018]. Reference Source

[30] NPA Responds to NAS’ The Postdoctoral Experience Revisited.2014[cited 12 Jul 2018]. In: National Postdoctoral Association. Reference Source

[31] Postdoc Policy Issues - A Brief Overview.In: National Postdoctoral Association. [cited 13 Jul 2018]. Reference Source

[32] Gardner L: Want to Kill Tenure? Be Careful What You Wish For. In: Chronicle of Higher Education.2018[cited 12 Jul 2018]. Reference Source

[33] Mathur A Lean SF Walker NV: Career outcomes for STEM, social and behavioral sciences and education doctoral alumni. *Ageing Sci Ment Health Stud.* 2018;2(3):1–33. Reference Source

[34] Damarin SK: The mathematically able as a marked category. *Gender Educ.* 2000;12(1):69–85. 10.1080/09540250020418

[35] Stayart AC Monsalve G Brandt PD: Applying inter-rater reliability to improve consistency in classifying PhD career outcomes - Extended Data S1-S11.2020 10.17605/OSF.IO/5CGUJ PMC7014580 32089837

