# Supplementary material for: Applying inter-rater reliability to improve consistency in classifying PhD career outcomes
Source: F1000Res. 2020 Apr 28;9:8. Originally published 2020 Jan 9. [Version 2] doi: 10.12688/f1000research.21046.2 (PMC7014580; doi:10.12688/f1000research.21046.2)
Supplement: Peer review history

# Peer review: Reviewer response for version 1

**DOI:** 10.5256/f1000research.23163.r58478

**Published:** 2020-02-10

**Authors:** Reinhart Reithmeier

**Affiliations:** 1 Department of Biochemistry, University of Toronto, Toronto, ON, Canada

This paper describes the results of an important pilot study using 800 alumni records to develop a uniform taxonomy to classify career outcomes of PhD graduates across institutions. Success in developing such a survey instrument will allow uniform reporting, aggregation of data and meaningful comparisons. Attempts were made to address ambiguities in some categories of the taxonomy, including the addition of a flag system. There still remained discordance among the coders even among those who went through a training exercise. The inter-rater reliability did not improve significantly from Experiment 1 to 2. In career outcomes there is the constant challenge to balance the use of broad categories and the degree of granularity necessary to generate meaningful data. 

 Comments:

1. In Table 1 the Workforce Sectors are broad: Academia, Government, For-Profit, Non-Profit, Other. This classification is based mainly on the nature of the **Employer **and should be fairly straight-forward to bin. Consider adding Self-employed. Workforce Sector would be a **Level 1 Bin**. 
2. The next level of classification within each Workforce Sector could be based on the **Job Title**, rather than Career Type and Job Function. This would be a **Level 2 Bin **and easy to code from drop-downs of typical job titles. A Job Title is easier to bin and often provides a good definition of the Job Function. A challenge that needs to be addressed are multiple job functions. In this regard Professor as a Job Title is an ideal career type as they all carry out three main job functions: teaching, research and administration all to various extents. Some Professors are also entrepreneurs and start companies. 
3. The use of Career Type: Primarily Research or Primarily Teaching at this level is not useful. Indeed, over a career trajectory the mix of teaching, research and administration for a Professor may change, yet the Career Type and does not. Determining whether a professor is primarily Research or Primarily Teaching could be difficult to determine, even based on the Carnegie classification of Institutions. Similarly, in the Private Sector the Job Title: Research Scientist could describe a dynamic mix of research and administration typical of people working in this sector. Placing individuals in the proper Job Titles using pre-populated drop-down menus should be straight-forward.
4. The current Table 3 Job Function is a muddle of job titles and job functions. I would not put Deceased and Retired under the same function!
5. The **Level 3 Bin **could now be Job Function under Job Title. This could gleaned from a search of the LinkedIn profile for pre-determined key words. Here, job functions such as Research, Teaching or Administration could be useful. Using the term *primarily *may be difficult to assess, other than what is already obvious for the Job Title (e.g., Manager, Director, V-P, etc.).
6. Further Training or Education should include Post-doctoral Fellow and Professional Schools as the two main categories where PhDs go.
7. The average time to classify at under 2 minutes seems very short.
8. The goal of this project involves the classification of thousands upon thousands of PhD alumni (big data) from institutions across the USA and is an ideal application of AI through machine learning to find meaningful patterns and classifications into bins. It would be useful to compare the results of the current project with the results obtained by computer neural networks using the same training data set. 

Is the work clearly and accurately presented and does it cite the current literature?

Yes

If applicable, is the statistical analysis and its interpretation appropriate?

Yes

Are all the source data underlying the results available to ensure full reproducibility?

Yes

Is the study design appropriate and is the work technically sound?

Yes

Are the conclusions drawn adequately supported by the results?

Yes

Are sufficient details of methods and analysis provided to allow replication by others?

Yes

Reviewer Expertise:

Graduate Professional Development, Teaching, Research

I confirm that I have read this submission and believe that I have an appropriate level of expertise to confirm that it is of an acceptable scientific standard.

# Response

**Published:** 2020-04-21

**Authors:** Rebekah Layton

**Affiliations:** UNC-Chapel Hill Office of Graduate Education, USA

The authors wish to thank our reviewers for their thoughtful comments and thorough review. Please find a point-by-point response below. R denotes reviewer comments, A denotes author responses.

**​​​​​**---

**R1: Reviewer 1 - Overall Comment:** “This paper describes the results of an important pilot study using 800 alumni records to develop a uniform taxonomy to classify career outcomes of PhD graduates across institutions. Success in developing such a survey instrument will allow uniform reporting, aggregation of data and meaningful comparisons. Attempts were made to address ambiguities in some categories of the taxonomy, including the addition of a flag system. There still remained discordance among the coders even among those who went through a training exercise. The inter-rater reliability did not improve significantly from Experiment 1 to 2. In career outcomes there is the constant challenge to balance the use of broad categories and the degree of granularity necessary to generate meaningful data.”

**A1: Author Response to Reviewer 1 - Overall Comment:** We thank the reviewer for a thoughtful and well-considered response. Although the gains in reliability were not dramatic, the reliability increased in every tier using the latter version (UCOT Exp2); more importantly, each of these tiers then met generally-accepted criteria for adequate reliability (at least with experienced coders). As noted, we agree that it is important to balance detail versus usability (granularity vs. breadth). We believe that UCOT Exp2 provides an example of how even slight modifications to wording and training can affect interpretation and reliability of the taxonomy, and that our experimental data demonstrate the necessity of further evolution of taxonomies as the workforce itself evolves.

**R1.1: **“In Table 1 the Workforce Sectors are broad: Academia, Government, For-Profit, Non-Profit, Other. This classification is based mainly on the nature of the Employer and should be fairly straight-forward to bin. Consider adding Self-employed. Workforce Sector would be a Level 1 Bin.”

**A1.1:** In response to the reviewer’s comment regarding Workforce Sector organization and inclusion of self-employment therein, we agree that self-employment is not accurately captured in UCOT Exp2 and that, since self-employment is becoming a more frequent career outcome for PhD-trained scientists, we must identify a way to represent it in the taxonomy. During our experiment, we did in fact consider the importance of self-employment, but realized that every form of self-employment could be classified within an existing sector of the taxonomy. For instance, the Workforce Sector a self-employed individual could be for-profit or non-profit (Tier 1), and, within that, their Job Type could be in any type (e.g., focused on teaching, research, or science-related; Tier 2), and finally, the nature of their self-employment would be appropriately captured in Job Function (Tier 3) as Entrepreneur. In our second iteration of the experiment (UCOT Exp2), we advised that self-employed individuals be classified in the field of their specialization (if freelance) or as entrepreneur (in Tier 3). However, that solution inadequately captures the important distinction of self-employment.

 Prompted by your comment, we have considered how self-employment could be introduced to the taxonomy while still satisfying taxonomic logic, since if a record can be classified within multiple categories in the same tier, an additional level of classification is required to capture the specialization of the record. We propose that, in Job Function, self-employed individuals continue to be classified by the field in which they are working (for example, communications), and that their self-employment characteristic could be captured by a flag system, similar to that used with faculty and postdoctoral scientists. We have now incorporated this as a recommendation in future directions.

 The following has been added to the text: “This iteration of the career taxonomy does not adequately capture the characteristic of self-employment, since self-employed individuals are currently classified by the field in which they are working. When the taxonomy was developed, the original UCOT designers agreed that when a classification could fall within multiple categories in the same tier, it should be considered subsequently in a different tier. In future variations, self-employment could be captured by an additional flag system similar to faculty or postdocs, which may be relevant to institutions and programs with an interest in entrepreneurship startups, and spinoffs.

**R1.2 & 1.5: **“The next level of classification within each Workforce Sector could be based on the Job Title, rather than Career Type and Job Function. This would be a Level 2 Bin and easy to code from drop-downs of typical job titles. A Job Title is easier to bin and often provides a good definition of the Job Function. A challenge that needs to be addressed are multiple job functions. In this regard Professor as a Job Title is an ideal career type as they all carry out three main job functions: teaching, research and administration all to various extents. Some Professors are also entrepreneurs and start companies….”

 “…The Level 3 Bin could now be Job Function under Job Title. This could gleaned from a search of the LinkedIn profile for pre-determined key words. Here, job functions such as Research, Teaching or Administration could be useful. Using the term primarily may be difficult to assess, other than what is already obvious for the Job Title (e.g., Manager, Director, V-P, etc.).”

**A1.2& 1.5:** Thank you for your thoughtful consideration of the structure of the taxonomy. We fully agree that starting with job titles seems straight forward and, historically, many career taxonomies started from that point, including our own. However, when applied to thousands of records by multiple individuals, we found an absence of convergence upon “typical” job titles due to the rapidly diversifying career pathways pursued by PhD-trained scientists. Indeed, it was the proliferation of job titles and their selective redefinition among industries that led to our observation that initial classification by job title actually contributed to inconsistent classification. This resulted in our proposal of an experiment in which we tested how well multiple independent coders could bin the same job title; we found that overreliance on job title was associated with unreliable coding (see V1, P13 Description of Changes to UCOT2017 for further discussion).

**R1.3:** The use of Career Type: Primarily Research or Primarily Teaching at this level is not useful. Indeed, over a career trajectory the mix of teaching, research and administration for a Professor may change, yet the Career Type and does not. Determining whether a professor is primarily Research or Primarily Teaching could be difficult to determine, even based on the Carnegie classification of Institutions. Similarly, in the Private Sector the Job Title: Research Scientist could describe a dynamic mix of research and administration typical of people working in this sector. Placing individuals in the proper Job Titles using pre-populated drop-down menus should be straight-forward.

**A1.3:**  Our key reason for classifying career type as research/teaching/science-related is the important implications for the types of career and professional development activities that could be pursued by trainees during the training period in order to be best prepared for that type of career. For example, PhD students may not need to pursue postdoctoral training if their intended career does not directly involve research, or those who are interested in primarily-teaching careers should pursue additional training in educational pedagogy and classroom teaching. Similarly, we have observed a tendency among faculty and trainees alike to assume that a professorial job title implies that the individual bears the research responsibilities of a Principal Investigator. By relying on job titles alone as a primary classification strategy, we sacrifice valuable detail regarding the actual function of faculty positions across the types of universities and colleges. We feel that failing to differentiate ‘primarily research’ from ‘primarily teaching’ does a disservice to the career development of trainees, and thus it is important to capture the primary work activity of the individual.

 Finally, many institutions and funding agencies place value in distinguishing between career outcomes that could be classified as ‘research-focused’ roles in higher education versus those that are ‘teaching-focused.’ Hence, we believe that this distinction is worth including and aids in interpretation and display of the data.

**R1.4:** The current Table 3 Job Function is a muddle of job titles and job functions. I would not put Deceased and Retired under the same function!

**A1.4:** We recognize the often-crucial role that experienced retirees can play in organizations, whether formal or informal. However, with regards to the functional use of the career taxonomy, the commonality between retirees and deceased individuals is the removal of their formal full-time effort from the economy. We group them together to distinguish them from individuals who are “Unemployed or Seeking Employment,” because the latter contribute to the unemployment rate for scientists. Nonetheless, if someone were interested in additional granularity, more detail could be tracked in institutional databases to distinguish between the two.

**R1.6:** “Further Training or Education should include Post-doctoral Fellow and Professional Schools as the two main categories where PhDs go.”

**A1.6:** We struggled with the decision to separate postdoctoral positions from ‘further training or education’ because a postdoctoral period is one during which the individual is obtaining further training in a specific career. However, upon reflection, we concluded that a postdoctoral training period is actually a blended role of trainee and employee, which primarily consists of learning on-the-job rather than formal coursework. Hence, in our view, postdoctoral fellowship shares more in common with employment than education. While postdoctoral training is also a learning period, we make a further distinction in our definitions that inclusion in “Further Education and Training” connotes the completion of coursework towards a formal degree or certification.

 Furthermore, by adopting postdoctoral trainees as a Job Function (acknowledging that it is with the dual role of training), we are able to maintain an important level of granularity to the ever-neglected category of postdoctoral trainee, whose roles often remain unexamined due to their complexity. In accordance with the reporting requirements of some national organizations (e.g., CNGLS), currently many institutions choose only to report Tiers 1 & 2. Given the probability of use of the taxonomy without including Tier 3- and given the increased availability of non-academic and non-research postdoctoral positions in areas such as policy, communications, teaching, industry, and more – we felt the visibility of non-traditional training roles would otherwise be compromised, leaving them for all intents and purposes, invisible.

 Due to the formal “Further Training or Education” definition adopted, and the need to capture different types of postdoctoral training, we remain committed to this structural change from previous versions, despite the controversial nature of this recommendation (see p12, “Description of Changes Made to Increase UCOT Reliability” for additional discussion). Regardless of any group’s decision for classifying postdoctoral fellows, we strongly advise taxonomy users to provide transparency and consistency of definition within appropriate context (e.g., if reporting for CNGLS, clearly label the version being reported). This may at times result in multiple sets of reports depending upon the number of taxonomic tiers an institution chooses to report, however we hope that over time, as institutions build baseline career outcomes data, it will become easier to update existing data sets with greater levels of detail if greater granularity if so desired.

**R1.7:** “The average time to classify at under 2 minutes seems very short.”

**A1.7:** We were also pleased with the very reasonable time per record, as averaged over multiple coders who each tracked time spent per number of records to provide this information (for further discussion see V1, P10, Results Section). We would like to clarify that this time reflects only the actual classification of the record, not the time required to find the individual. We also underline that all coders used in our study were PhD-trained program directors in graduate education, which imbues a bias of familiarity with career outcomes, job titles, and job functions that are typical among PhD-trained scientists. In future directions, we suggest that comparisons of time and accuracy produced by other types of coders should be explored, and that, in all cases, teams should be trained as a unit to achieve maximum concordance within the unit.

**R1.8:** “The goal of this project involves the classification of thousands upon thousands of PhD alumni (big data) from institutions across the USA and is an ideal application of AI through machine learning to find meaningful patterns and classifications into bins. It would be useful to compare the results of the current project with the results obtained by computer neural networks using the same training data set.”

**A1.8:** The authors concur that this would be an interesting iteration for future studies to explore. As insights from big data have transformed much of our current economy and society, there are most certainly interesting applications for use of machine learning and big data to apply to PhD career outcomes. With that said, there are limitations to technological applications in their ability to distinguish nuances between these categories or consider situational differences. Future experiments could use machine learning to develop a taxonomy de novo, based in its own determination of which parameters, definitions, searches, etc. would create the taxonomic tiers. Indeed, applying a neural networking model to generate a career taxonomy would be fascinating! In the interim, comparisons of human vs. machine performance, or hybrid human/computer coding might be a good initial exploration.

 In response to the reviewer’s comment, we have introduced the following text to our manuscript: “While this experiment focused on evaluating the consistency and time investment needed for manually classifying career outcomes, future directions to improve efficiency might include development of automated systems or programs to implement career classifications, particularly with the advent of machine learning in the current era. As a first step, perhaps machine-learning could increase efficiency by creating initially proposed binning of career outcomes which could then be spot-checked or confirmed by a human being on a second pass. Perhaps this process could even be fully automated pending technological advances.”

# Peer review: Reviewer response for version 1

**DOI:** 10.5256/f1000research.23163.r58480

**Published:** 2020-01-23

**Authors:** Chris Pickett

**Affiliations:** 1 Rescuing Biomedical Research, Lewis-Sigler Institute, Princeton University, Princeton, NJ, USA

The article “Applying inter-rater reliability to improve consistency in classifying PhD career outcomes” by Stayart *et al.* examines how consistent different raters are when making taxonomic determinations of Ph.D. career types. Starting with a taxonomy developed by several groups and universities in 2017, the authors test the degree of agreement among six different coders classifying several hundred records. They then identified areas of disagreement among the coders and made critical definitional changes to the taxonomy that improve the degree of agreement across coders.

 The paper by Stayart *et al.* is an important contribution to the effort to encourage universities to be more transparent about the careers their Ph.D.s take after they graduate. Improving the career taxonomy and strengthening the definitions therein, along with the authors’ discussion of the shortcomings of these taxonomic approaches, will make it easier for more institutions to transparently provide this kind of information to their trainees.

 Major issue:

- The text in the Discussion under the “Description of changes made to UCOT 2017 to increase reliability” reads as results rather than discussion. The authors should strongly consider moving this entire section into the Results section to help readers understand the real differences between UCOT Exp 1 and UCOT Exp 2 as well as the beneficial changes the authors made to their final taxonomy.

Minor issues:

- “…a major impeding factor in this effort to share outcomes data has been the multitude of career outcomes taxonomies…” is not quite correct. The impediment, in my view, is not the taxonomies per se, but the small, yet important, differences in how career outcomes are binned, leading to real differences in taxonomies and data presentation. The authors echo this point of view in the Discussion when they caution institutions not about making new taxonomies, but about being forthright with any changes they make to the common taxonomy. The authors should consider recasting this sentence.
- In the third paragraph of the Introduction, it may be useful to the reader to include an example of a job title that could potentially fall into more than one category.
- A basic diagram of the three tiers of UCOT 2017 as Fig. 1 may help readers understand how the scheme works and provide a firmer basis for understanding the experiments that follow. The authors present the three tiers in three separate tables, but it is difficult to understand from these tables how the tiers function together.
- It would be useful for the authors to provide information on the degree of agreement among the coders after the second experiment. After Exp1, the authors remark “The six coders in Experiment 1 had six-way agreement on 77% of all records within…” What percentage of records were coded the same by the coders in Exp2, and what were the areas of disagreement? Were the problems areas identified in Exp1 resolved in Exp2?
- The authors should take a little more space to discuss the issue of time needed to code records. The authors report it takes coders about 1 min 40 seconds per record to code them; however, the average time for experienced and inexperienced coders comes out to be about a 30-second per record difference (328 min/219 records =~1.5 min; 429 min/219 records =~2 min). At this level of time, that’s an important difference!

Is the work clearly and accurately presented and does it cite the current literature?

Yes

If applicable, is the statistical analysis and its interpretation appropriate?

Yes

Are all the source data underlying the results available to ensure full reproducibility?

Yes

Is the study design appropriate and is the work technically sound?

Yes

Are the conclusions drawn adequately supported by the results?

Yes

Are sufficient details of methods and analysis provided to allow replication by others?

Yes

Reviewer Expertise:

Science policy

I confirm that I have read this submission and believe that I have an appropriate level of expertise to confirm that it is of an acceptable scientific standard.

# Response

**Published:** 2020-04-21

**Authors:** Rebekah Layton

**Affiliations:** UNC-Chapel Hill Office of Graduate Education, USA

The authors wish to thank our reviewers for their thoughtful comments and thorough review. Please find a point-by-point response below. R denotes reviewer comments, A denotes author responses.

 ---

**R2: Reviewer 2 - Overall Comments:**

 “The article “Applying inter-rater reliability to improve consistency in classifying PhD career outcomes” by Stayart et al. examines how consistent different raters are when making taxonomic determinations of Ph.D. career types. Starting with a taxonomy developed by several groups and universities in 2017, the authors test the degree of agreement among six different coders classifying several hundred records. They then identified areas of disagreement among the coders and made critical definitional changes to the taxonomy that improve the degree of agreement across coders.”

 “The paper by Stayart et al. is an important contribution to the effort to encourage universities to be more transparent about the careers their Ph.D.s take after they graduate. Improving the career taxonomy and strengthening the definitions therein, along with the authors’ discussion of the shortcomings of these taxonomic approaches, will make it easier for more institutions to transparently provide this kind of information to their trainees.”

**A2: Authors response to Reviewer 2 - Overall Comments:** We thank Reviewer 2 for noting the importance of developing a culture of transparency around career outcomes, to which we hope this paper contributes. The authors main purpose in executing the iterative experiment was, indeed, to ultimately serve the practitioner responsible for coding the information. We also believe that the benefit of more reliable and consistent classifications will trickle down to important stakeholders, including prospective and current trainees, who will consequently be empowered with high-quality data to confidently make their own informed career decisions. Furthermore, policy changes are often instigated by data – without robust, reproducible, and reliable data, it is difficult to advocate for evidence-based policy changes around career and professional development.

**R2.1. Major: **“The text in the Discussion under the “Description of changes made to UCOT 2017 to increase reliability” reads as results rather than discussion. The authors should strongly consider moving this entire section into the Results section to help readers understand the real differences between UCOT Exp 1 and UCOT Exp 2 as well as the beneficial changes the authors made to their final taxonomy.”

**A2.1 Major:** Thank you for this excellent suggestion, we agree that this would strengthen the flow and readability of the paper without sacrificing content. We have adopted this change as recommended and moved this section into the results.

**R2.2 Minor issues: **“…a major impeding factor in this effort to share outcomes data has been the multitude of career outcomes taxonomies…” is not quite correct. The impediment, in my view, is not the taxonomies per se, but the small, yet important, differences in how career outcomes are binned, leading to real differences in taxonomies and data presentation. The authors echo this point of view in the Discussion when they caution institutions not about making new taxonomies, but about being forthright with any changes they make to the common taxonomy. The authors should consider recasting this sentence.

**A2.2:** Thank you for sharing this helpful perspective. We have changed the wording to reflect this suggestion. We changed the original sentence from, “However, a major impeding factor in this effort to share outcomes data has been the multitude of career outcomes taxonomies developed by individual institutions and agencies.”- To: - “However, a factor that has impacted the sharing of outcomes data has been a confusion over which career outcomes taxonomy is being displayed in which context.”

**R2.3:** “In the third paragraph of the Introduction, it may be useful to the reader to include an example of a job title that could potentially fall into more than one category.”

**A2.3: **Thank you for pointing out the need for an example here, we agree that this would strengthen the text and have added the following accordingly: **“**For example, a Program Director title at a University could have very different job responsibilities compared to a person of the same title in a life science consulting firm.”

**R2.4 Minor issues: “**A basic diagram of the three tiers of UCOT 2017 as Fig. 1 may help readers understand how the scheme works and provide a firmer basis for understanding the experiments that follow. The authors present the three tiers in three separate tables, but it is difficult to understand from these tables how the tiers function together.”

**A2.4:** We agree that a figure with a broad level overview of how the tiers fit together would be helpful to the reader. In our attempt to do this in the past we found it difficult to maintain fonts that were readable in a small amount of space. Hence, we were unable to create a visual display that fit effectively on a manuscript page to illustrate this. Furthermore, in order to represent all variations, we would need multiple iterations of the figure, which may not be a good use of space (duplicative), or worse yet, could be confusing as to which variant is being discussed. Hence, after repeated failed attempts, we concluded that an effective visualization was not feasible.

**R2.5 Minor issues: **“It would be useful for the authors to provide information on the degree of agreement among the coders after the second experiment. After Exp1, the authors remark “The six coders in Experiment 1 had six-way agreement on 77% of all records within…” What percentage of records were coded the same by the coders in Exp2, and what were the areas of disagreement? Were the problems areas identified in Exp1 resolved in Exp2?

**A2.5:** We explored using ‘coder-agreement’ as a metric, however concluded that there were  inherent limitations of this method and elected to use a more robust measure of coder agreement, Krippendorf’s alpha. Using this more sophisticated measured, we hoped to be able to compare it to acceptable levels of agreement as established and validated in previous literature (unrelated to taxonomies). Furthermore, use of Krippendorf’s alpha avoids inherent flaws in the simpler ‘coder-agreement’ metric, such as potentially overestimating agreement by not taking ‘agreement by chance’ into account. We believe that Krippendorf’s alpha permitted a more robust comparison across both studies.

**R2.6 Minor issues: **“The authors should take a little more space to discuss the issue of time needed to code records. The authors report it takes coders about 1 min 40 seconds per record to code them; however, the average time for experienced and inexperienced coders comes out to be about a 30-second per record difference (328 min/219 records =~1.5 min; 429 min/219 records =~2 min). At this level of time, that’s an important difference!”

**A2.6:** Based on the reviewer’s suggestion, we have addressed in greater detail what this would mean in real time for experienced versus inexperienced coders, and added the following example to the text:

 “The total time for experienced coders ranged from 213 to 540 minutes, on average 328 minutes, or about a minute and a half. In comparison, the total time for inexperienced coders ranged from 246 to 545 minutes coders, on average 429 minutes, or about two minutes. While this may not seem like a huge difference in time to code records for inexperienced versus experienced coders, depending on the size of one’s alumni populations, this may affect one’s approach. For instance, if binning 1000 records, taking the time to become a trained “experienced coder” would save each coder approximately a day’s worth of full time effort (e.g., approximately 33 hours versus 25 hours).”
